# Supplementary material for: Polyketide synthase gene clusters in the ericoid mycorrhizal fungus Oidiodendron maius: genome-wide prediction, expression profiling, and functional characterization of knockout mutants
Source: Front Plant Sci. 2026 Sep 16;17:1924430. doi: 10.3389/fpls.2026.1924430 (PMC13623903; doi:10.3389/fpls.2026.1924430)
Supplement: Supplementary file 1 [file SupplementaryFile1.zip › Supplementary Materials/Supplementary Folder 1.zip!/Oidma1_AssemblyScaffolds_Repeatmasked/knownclusterblast/cluster33/jgi.p_Oidma1_169142_mibig_hits.html]

| MIBiG Protein | Description | MIBiG Cluster | MiBiG Product | % ID | % Coverage | BLAST Score | E-value |
| --- | --- | --- | --- | --- | --- | --- | --- |
| AGN71609.1 | amino\_oxidase/esterase | BGC0000027 | Azaphilone\_biosynthetic\_gene\_cluster | 36 | 101.545253863 | 265 | 1.1e-70 |
| BAK26560.1 | FAD-dependent\_oxidoreductase | BGC0000977 | Cyclopiazonic\_acid\_biosynthetic\_gene\_cluster | 30 | 101.766004415 | 205 | 1.8e-52 |
